# Examining the influence of lifestyle variables on the accuracy of skeletal age estimation via the pubic symphysis

**DOI:** 10.1111/1556-4029.70240

**Published:** 2025-11-28

**Authors:** Natalie Moss, Elizabeth Craig‐Atkins

**Affiliations:** ^1^ University of Sheffield Sheffield UK; ^2^ Present address: University of Cambridge Cambridge UK

**Keywords:** age estimation, forensic anthropology, New Mexico Decedent Image Database, pubic symphysis, random forest, transition analysis

## Abstract

This study investigated links between skeletal age estimation error and lifestyle variables to better elucidate sources of interpersonal variability in the rates of skeletal aging. Skeletal age for 180 individuals from the New Mexico Decedent Image Database was estimated by applying the Suchey–Brooks method and transition analysis to 3D models of the pubic symphysis, and age estimates were compared to known age‐at‐death. Age estimation bias and accuracy for both methods were evaluated first with respect to single lifestyle variables, then random forest modeling was used to test variability with respect to all lifestyle variables. Age estimation bias was shown to be significantly different with respect to sex when applying transition analysis, but not when applying Suchey–Brooks, and males tended to be underaged relative to females of the same age. While no statistically significant differences in bias existed for either method between BMI categories, random forest modeling indicated that body size exerts a limited but variable influence on skeletal aging. Additional variables were highlighted as potentially influential to skeletal aging by random forests, such as socioeconomic status, but ultimately, model performance and variable importance plots demonstrated that these influences were slight and nonuniform. These data suggest that including considerations of lifestyle variables in skeletal aging methods would not improve aging estimates.

**Highlights**

- Transition analysis may underage older males relative to females when analyzing the pubic symphysis.
- Body size and pregnancy explain a limited amount of variability in skeletal aging.
- Other lifestyle variables may also contribute minimally to skeletal aging.
- It is neither necessary nor feasible to incorporate lifestyle variables in forensic age estimation.

## 1 INTRODUCTION

Adult age estimation from the human skeleton results in fairly broad age estimates, which can limit their utility in bioarchaeological and forensic investigations. In children and adolescents, skeletal age can be estimated precisely and accurately due to relatively constrained time frames for epiphyseal fusion and tooth eruption [1, 2, 3, 4]. However, beyond approximately 30 years of age when skeletal growth and fusion are effectively complete, age estimates are reliant on degenerative changes, which do not proceed at well‐established rates and vary substantially between individuals [5, 6, 7]. The discordance between true chronological age and estimated skeletal age therefore necessitates the construction of broad age ranges [6, 7, 8, 9]. Broad ranges allow for the use of estimation methods that achieve high success rates in capturing an individual's age‐at‐death, but this success is contingent on imprecision. This is the crux of the major issue in skeletal age estimation: adult age ranges cannot be both precise and accurate [1, 10, 11, 12, 13, 14, 15].

Imprecise age estimates lead to the overall homogenization of demographic profiles. Younger adults are often overaged, whereas older adults are consistently underaged [9, 12]. In forensic anthropology, where estimating age‐at‐death aids in the identification of unknown decedents [12, 16, 17, 18], a lack of precision may lead to oversights in correlating missing persons records to unidentified individuals [15, 17].

Applications of advanced mathematical modeling have shown promise in resolving reference sample mimicry. Boldsen et al. [1] introduced transition analysis, an application of Bayes' Theorem, to age estimation based on the scoring of age‐related features on the pubic symphysis, auricular surface, and cranial suture closure. Transition analysis uses estimates of trait probabilities and is less biased by the reference samples used in its construction [14]. Its Bayesian approach has been shown to outperform traditional morphological aging methods, such as the widely applied Suchey–Brooks method [16, 19], although age ranges for older adults remain broad [1]. More recently, Navega et al. [20] outlined a machine learning approach to age estimation based on a deep randomized neural network. However, despite generating accurate estimates of age across the full lifespan, older individuals still retain broad ranges of error using both Bayesian and machine learning approaches [1, 7, 20].

The failures of mathematical models to sufficiently narrow age estimates in older adults are not due to flaws in their approach, but rather, inherent flaws in the data on which they rely. Many osteologists have adopted the attitude that it is realistically impossible to ever identify age reliably or precisely in older individuals due to the cumulative nature of aging over the lifecourse [14]. However, we still lack a fundamental understanding of *how* senescence proceeds in the skeleton, and there has been comparatively little effort to understand the exact reasons for its high degree of interpersonal variability. While similar potential confounding variables are commonly discussed, including socioeconomic status, body size, population genetics, activity level, and environment [5, 8, 12, 13, 15, 18, 21], research investigating the real influence of these variables is scarce. The exception is body size, which has been the subject of research by Merritt [17, 18, 22] and Wescott and Drew [23]. This situation partially reflects the lack of lifestyle metadata associated with most skeletal populations. The establishment of modern skeletal data repositories, however, has provided an opportunity to revitalize the exploration of the impact of confounding variables on skeletal age assessment as a necessary accompaniment to advances in statistical handling of age data [18, 21].

The present study evaluates discrepancies in skeletal aging error in a forensic population, in order to seek relationships between chronological age at death, skeletal age, and various potential confounding variables. In doing so, it presents a novel and more nuanced understanding of how lifestyle variables contribute to the rate of skeletal aging and evaluates the necessity of accounting for lifestyle variables in forensic age estimates.

## 2 MATERIALS AND METHODS

### 2.1 Sample population and methods selection

The New Mexico Decedent Image Database (NMDID) was identified as a suitable population on which to test the impact of confounding variables on skeletal aging. NMDID contains high resolution, full‐body postmortem CT scans for over 15,000 individuals who died in New Mexico between 2010 and 2017 [24]. Scans are optimized for bone, and are associated with each anonymized individual alongside reported lifestyle metadata and medical histories [24]. The sample population was limited to individuals with available data on biological sex, chronological age, ancestry, chronic physical and mental illness, birthweight category, reported dietary pattern, reported exercise and strenuous lifting, adult and childhood socioeconomic status, tobacco use, habitual drinking patterns, pregnancies, body size (living height, living weight, and BMI), and the use of illicit substances. These variables encompass potential accelerants of skeletal senescence that have been previously mentioned in the literature [5, 8, 12, 13, 15, 18, 21], and function as proxies for physiological stress and genetic variation. A random sample of 180 adult individuals ranging in age from 22 to 85 years was selected from the NMDID database (data included in Table S1). The final sample included 111 males and 69 females, and the average chronological age of the sample was 50.7 years.

While 3D reconstructions do not contain the same level of detail as physical skeletal material, previous research has successfully integrated clinical data into studies of skeletal aging by relying on 3D CT imaging. Wink [25], Lottering et al. [26], and Merritt [18, 27] have all employed CT scans in aging studies of the pubic symphysis. The Suchey–Brooks method, first published in 1990 [19], remains one of the most popular ways of estimating age from the pubic symphysis [28, 29, 30]. While the Suchey–Brooks method has been shown to be less accurate in aging older adults than the revised aging method published by Hartnett [30], Suchey–Brooks was preferentially adopted in this study due to the nature of the CT‐based reconstructions and the need to incorporate the comprehensive associated metadata of NMDID into this project framework. The diagnostic criterion of Hartnett [30] relies in part on the physical weight of the pubis, which cannot be ascertained from reconstructions. Further, the Suchey–Brooks method analyzes many of the same age‐related features as Hartnett, and has been successfully applied to previous CT‐based studies [18, 25, 26, 27].

The Suchey–Brooks method relies on sorting skeletal elements into phases based on their observable morphology [19]. Each phase has an age range, average age, and reported standard deviation, which reflect phase‐specific age distribution in the original reference sample [19]. Application of this method to an unknown decedent yields a pubic symphysis stage I–VI, which corresponds to two different sets of age estimates for males and females.

Transition analysis (TA) was additionally used to age all individuals in order to better understand the relationship between confounding variables, resultant age estimates, and specific mathematical models. Individual components of the pubic symphysis (e.g., symphyseal relief, ventral margin, and superior protuberance) are evaluated and scored independently, then input into the ADBOU program [31] (version 2.1.046, available at https://www.statsmachine.net/software/ADBOU2/), which also accounts for both sex and ancestry. ADBOU scores similar features on the pubic symphyseal surface to Suchey–Brooks then applies TA, adopting a different approach to generating final age estimates: Suchey–Brooks is based on observed age distributions, whereas ADBOU relies on a Bayesian distribution [32]. ADBOU provides a point estimate of age, which represents the maximum likelihood estimate for the age of that individual [33], rather than the phase‐specific average age reported for Suchey–Brooks. A 95% confidence interval for the age estimate is additionally generated by ADBOU based on skeletal data from the Terry and Coimbra reference collections [33]. The Suchey–Brooks method is therefore phase‐specific, whereas ADBOU relies on the evaluation of individually scored components to synthesize its age estimate [19, 33]. By integrating both Suchey–Brooks and TA, this project aimed to clarify if skeletal age markers are measurably affected by potential confounding variables irrespective of the mathematical approach to estimating age.

### 2.2 Scan processing

OsiriX, which has been employed by multiple research projects requiring a high level of CT reconstruction detail [18, 25, 34, 35, 36], was selected as the modeling software with which to conduct all 3D reconstructions. A 64‐bit open access version of OsiriX has been made available online under the name Horos. As such, Horos v3.3.6 (available through the GNU Lesser General Public License, version 3, at www.horosproject.org) was used for all reconstructions.

Prior to aging the pubic symphysis, full‐body CT scans were downloaded as DICOM files from NMDID, then imported to Horos for processing and modeling. All pubic‐symphyseal scoring was done while blind to chronological age, although biological sex and ancestry were known, as they were required for the most accurate application of aging methods. Following 3D reconstruction of the images in Horos, the “3D Volume Rendering” tool was selected for use from the “2D/3D” dropdown menu. The gradient icon was used to remove all soft tissue and reveal a 3D reconstruction of the skeleton, and the scissors icon was used to isolate pubic symphyseal surfaces from each other. Left surfaces were isolated first, then right.

**FIGURE 1:**
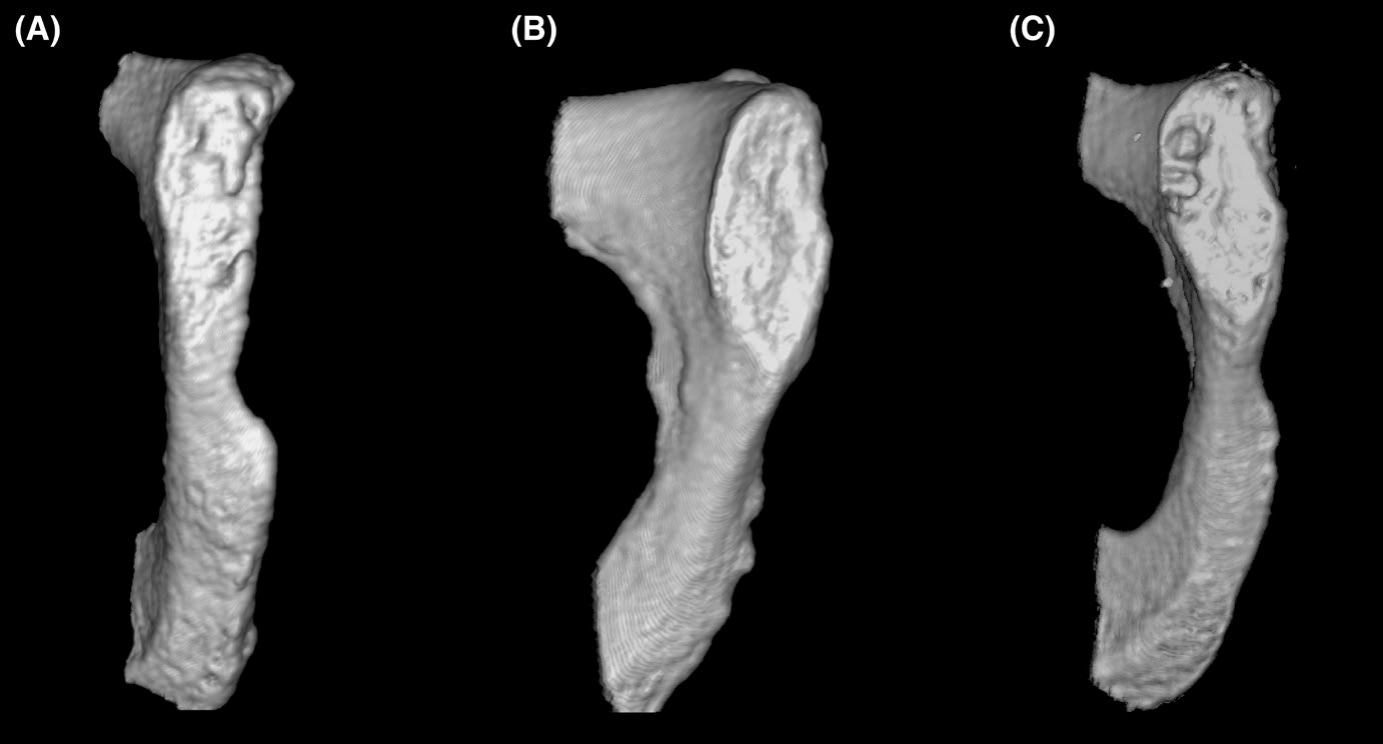
Reconstructed public symphyses. Example of three different pubic symphyseal faces modeled in Horos to show the level of reconstruction detail. From left to right: Pubic symphysis from a 22‐year‐old male, scored as a phase III; pubic symphysis from a 37‐year‐old male, scored as phase IV; and pubic symphysis from a 52‐year‐old male, sorted as phase VI. All symphyseal faces were manipulated in three‐dimensional space when scoring.

Symphyseal surfaces were manipulated in space in order to view the joint surface from multiple angles (Figure 1) and assigned a phase based on visual assessment of their morphology in accordance with Brooks and Suchey [19]. Features of the pubic symphysis were additionally scored according to guidelines accompanying ADBOU [31] and input into the program. Corresponding informative priors for individuals with ancestry reported as “white” or “black” were selected as described by Simon and Hubbe [32], but specific informative priors were not used for individuals who reported their ancestry as Hispanic, Native American, or mixed race. The “forensic” population box was additionally selected, as the majority of individuals in this sample had not died from natural causes [37]. This yielded point estimates of age and 95% confidence intervals for age estimation error based on prior distributions [31, 32, 37, 38].

All data, following initial recording in Microsoft Excel, was exported as a .csv and transferred to R Studio (Version 2024.09.0, Posit Software, PBC) for further analysis.

### 2.3 Data processing and statistical methods

In accordance with Merritt [22], Wescott and Drew [23], and Simon et al. [39], this study analyzed both aging *bias*, or the difference between a single‐value estimate of skeletal age and chronological age, and aging *accuracy*, or the success of the method‐specific age range in capturing the chronological age of an individual. Both left and right pubic symphyseal estimates were included in the data analysis, as differences in scoring between the left and right were minimal, and no consistent directional asymmetry in age estimates was observed.

Left and right Suchey–Brooks pubic symphyseal age estimates were first standardized by calculating the *z*‐scores of their aging bias: the chronological age of the individual was subtracted from the estimated age of the individual to find the bias for each pubis, and divided by the standard deviation (σ) of the assigned phase:

SkeletalAge–ChronologicalAge=Bias

Bias/Phase−Specificσ=Z−Score

Non‐standardized biases were also retained to evaluate relative patterning in age estimates among individuals. A rough holistic point estimate of aging bias was created for each individual by averaging left and right Suchey–Brooks biases:

$$\left(\text{Left Bias}+\text{Right Bias}\right)/2=\text{Holistic Point Bias}$$

Aging accuracy was also sorted into one of three categories for each individual: “range captures chronological age,” “underaged” or “overaged.” If the chronological age fell within the original age range reported for that phase, it was coded as “range captures chronological age.” If the chronological age fell below this range, the individual was considered “overaged,” and if the chronological age fell above the range, the individual was considered “underaged.”

TA point estimates of age were subtracted from chronological age to generate the TA aging bias for each individual. Aging accuracy was quantified on the same basis as Suchey–Brooks aging accuracy, using the provided TA ranges.

Consequently, three metrics of aging bias for the pubic symphysis were generated for each NMDID individual: standardized Suchey–Brooks aging bias, unstandardized Suchey–Brooks aging bias, and TA aging bias based on ADBOU.

This study aimed to first evaluate variation in aging bias with respect to single lifestyle variables using univariate methods. However, this analysis was limited, as only two lifestyle variables could be tested with respect to aging bias: biological sex and BMI category. The remainder of lifestyle variables did not demonstrate the same age distribution between different subsets of the variable: for example, individuals who were associated with “chronic physical illness” were significantly older than individuals who were not, and therefore, aging bias could not be compared between the two groups while also controlling for the expected and undisputed relationship between aging bias and chronological age.

As the distribution of age was not determined to be significantly different for males and females, or between the five BMI categories, Shapiro–Wilk tests of normality [40, 41] were conducted to determine if parametric or nonparametric methods should be employed to test differences in aging bias. *T*‐tests and Mann–Whitney *U*‐tests [42, 43] were then used to test differences in (a) standardized Suchey–Brooks aging bias, (b) unstandardized Suchey–Brooks aging bias, and (c) TA aging bias between males and females. One‐way ANOVA or Kruskal–Wallis tests [43] were then used to test differences in the same three measures of bias across different BMI categories.

Univariate analysis was also limited by the fact that lifestyle variables likely do not exert influence in isolation from each other. It was therefore necessary to integrate more complex models to investigate disparities between estimated age and chronological age. Random forest models were chosen based on their ability to handle both categorical and numerical data, and were used to test the impact of lifestyle variables on both age estimation bias and age estimation accuracy.

Random forests are a robust type of supervised machine learning that aggregates “forests” of decision trees to determine how accurately a parameter of interest can be modeled by a set of predictor variables, and the relative significance of each variable in constructing the model [44, 45, 46]. Decision trees have been previously integrated effectively in forensic anthropology for the estimation of ancestry [46] and biological sex [47], but in complex datasets, they are often prone to overfitting errors [44]. Random forests overcome these overfitting errors by constructing many trees, in which data is divided into subsets that are as homogenous as possible with respect to the variable of interest [48]. The remainder of the variables in the dataset, the predictor variables, are used to guide these data splits [48].

Random forest models are considered to be “ensembles,” in which all individually grown trees have been collectively analyzed to determine how accurately the data can be split [46]. For classification models (where the variable of interest is categorical), the out‐of‐bag error (OOB) describes how well the model performs [46, 49]. For regression models (where the variable of interest is numeric), the mean square error (MSE) and the coefficient of determination (*R* ^2^) are used to indicate model performance, which describes the variability between expected and observed values [48, 50]. Variable importance can be calculated by analyzing the effect of individual predictor variables on the overall error rate for classification trees, and on the MSE for regression trees [49]. Variable importance can also be quantified for classification trees by analyzing the effect of that predictor on the Gini index, a measure of node impurity [44, 46, 49].

Regression random forests were used to evaluate age estimation bias using the randomForest R package [49] (version 4.7‐1.2). Because random forests cannot contend with null values, three forests were grown for both unstandardized Suchey–Brooks bias and TA bias: one for males only, one for females only (including data on pregnancy), and one for both sexes (not including data on pregnancy). Individuals with missing height and weight data were also excluded, leading to a reduced sample size of 161. For each model, data were separated into training (75%) and testing (25%) data subsets. Models were evaluated based on the percentage of variance in aging bias they were able to explain, calculated from the MSE, as well as their *R* ^2^ value. Variable importance plots were also generated based on the impact of individual variables on decreasing the MSE [49].

Age estimation accuracy was then evaluated using classification random forests, based on the randomForest R package [49] and recommendations for model optimization outlined by Nikita and Nikitas [44]. The number of trees (*n*) and the number of predictors used in node splitting (*m*) resulting in the lowest out‐of‐bag (OOB) error were determined first, then used to train the best possible model, which was applied to the test dataset. Variable importance plots were also generated for all models on the basis of decreases in the mean model error and Gini impurity.

## 3 RESULTS

### 3.1 Univariate methods

**FIGURE 2:**
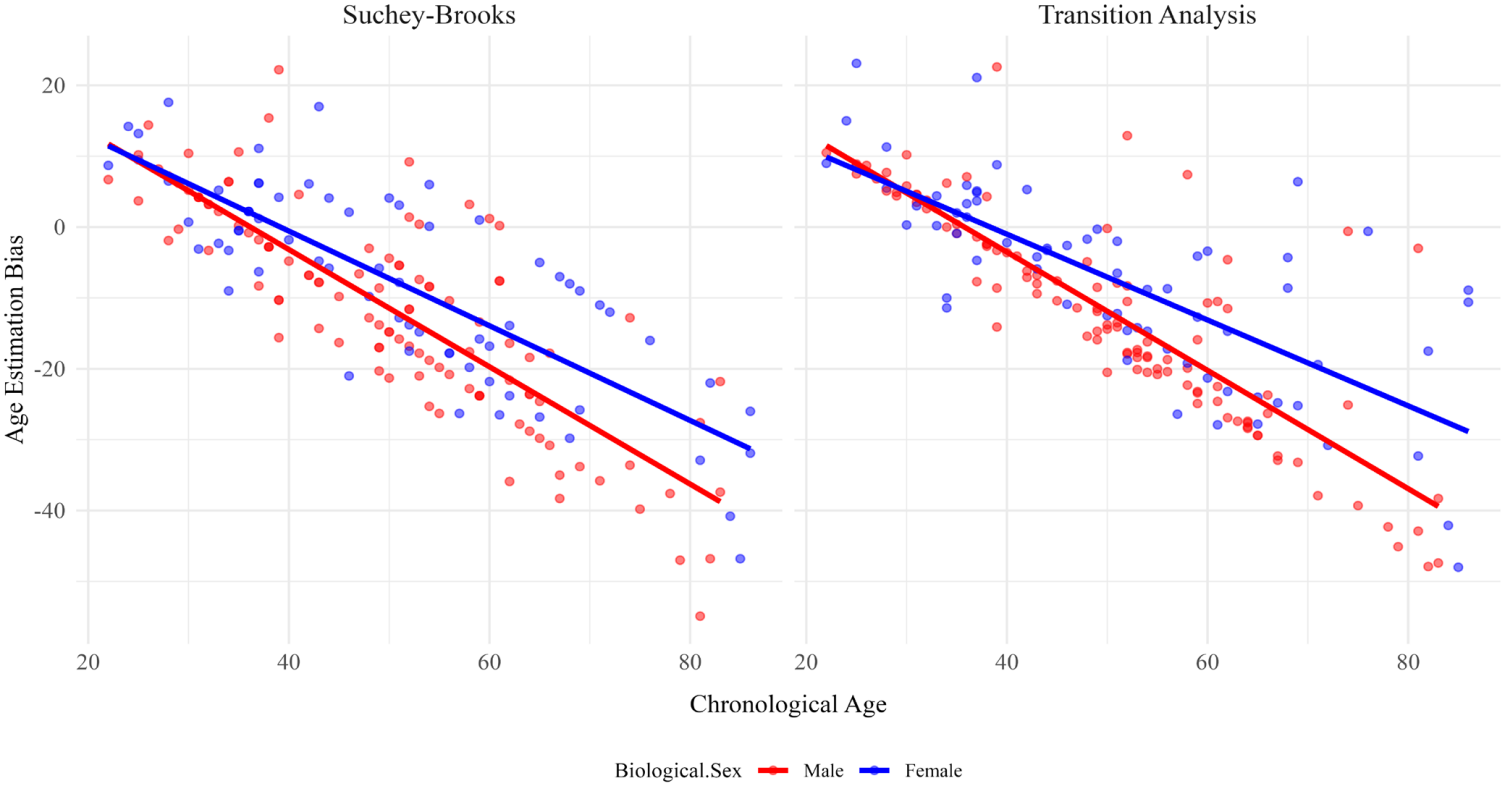
Age estimation bias by biological sex. Linear models of holistic age estimation bias in response to increasing chronological age, separated by male (red) and female (blue) data. Suchey–Brooks' age estimation bias is depicted on the left, and transition analysis age estimation bias is depicted on the right.

No statistically significant difference in age estimation bias was found between males and females when employing the Suchey–Brooks method. Neither left nor right *z*‐scores showed statistically significant differences in their distribution between males and females (Mann–Whitney *U*‐test, *p* = 0.10; *p* = 0.14). There was also no significant difference in the distribution of holistic aging bias between males and females when an independent samples *t*‐test was employed (*p* = 0.076). However, for ADBOU estimates, there was a statistically significant difference between the distribution of age estimation bias for males and females as evaluated by an independent samples *t*‐test (*p* = 0.035). Both male and female individuals tended to be underaged increasingly with age after approximately 40, but males were more underaged compared to females when scoring the pubic symphysis via transition analysis (Figure 2).

No statistically significant difference in aging bias distribution was found between different BMI categories for any metric analyzed. A one‐way ANOVA test was used to evaluate differences in left *z*‐score distribution between BMI categories, and returned a non‐significant *p*‐value of 0.26. For the right *z*‐scores, a Kruskal–Wallis test was employed, returning a non‐significant *p*‐value of 0.78. Distributions of holistic Suchey–Brooks aging bias and TA bias between BMI groups were also not significant when employing one‐way ANOVA and Kruskal–Wallis tests, respectively (*p* = 0.39; *p* = 0.58). Therefore, no difference in aging bias with respect to BMI was found for any age category when employing either Suchey–Brooks or TA.

### 3.2 Random forests

Random forest models generated using all available metadata for all individuals were illustrative of more complex potential interactions among lifestyle variables and skeletal age estimates.

Regression random forest models explained between approximately 49% and 54% of the total variance in Suchey–Brooks aging bias across the three population subsets tested (Table 1). Models generated for males only and for both sexes explained approximately the same amount of variance (53.93% vs. 53.59%), although the *R* ^2^ value was higher for the model including both sexes. The model generated for females explained slightly less of the variance (49.23%), but its *R* ^2^ value was approximately equivalent to the *R* ^2^ value for the model generated for males only.

**TABLE 1:** Regression random forest results.

Aging bias	Subset	% variance explained	*R*‐squared (test data)
Suchey–Brooks	Males only	53.93	0.514
	Females only	49.23	0.513
	Both	53.59	0.639
Transition analysis	Males only	55.28	0.671
	Females only	37.26	0.333
	Both	57.01	0.522

*Note*: Summary of regression random forest results, where model accuracy was evaluated on the basis of the amount of variance in aging bias that could be explained by the model, as well as the coefficient of determination, *R* ^2^.

**FIGURE 3:**
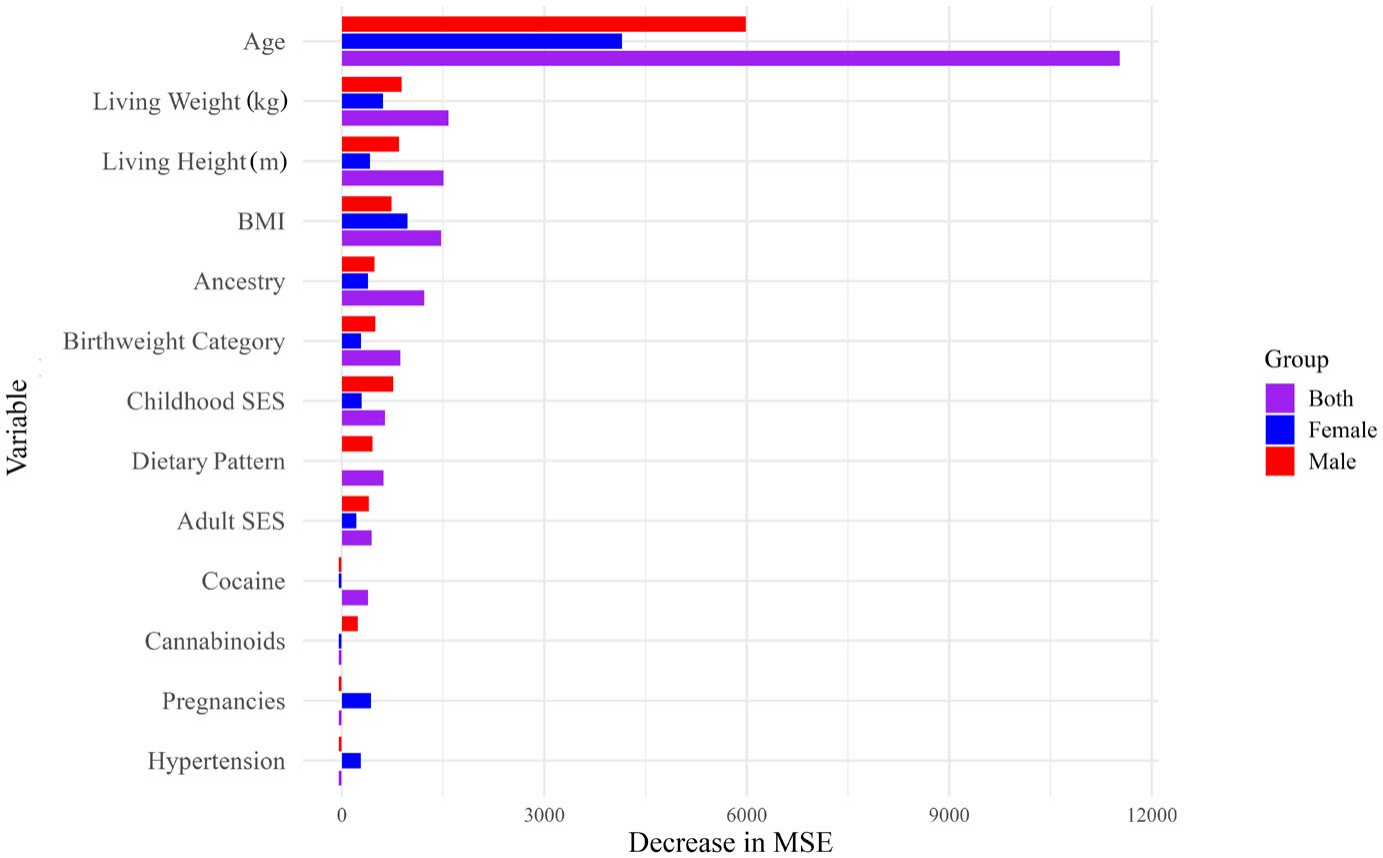
Regression variable importance comparison plot, Suchey–Brooks bias. Most important variables to the construction of the Suchey–Brooks regression forest models for both sexes (purple), males only (red), and females only (blue). Variables are ranked by the influence of that predictor variable on decreasing the overall mean square error (MSE).

All three variable importance plots demonstrated that “age” was by far the most important variable in the construction of the model. This was followed by metrics of body size: for both sexes and males only, it was “living weight” and “living height”; for females, it was “BMI” and “living weight” (Figure 3). “Childhood socioeconomic status” was ranked above BMI for males. “Pregnancies” was ranked above “living height” for females, and “living height” was also approximately as important as “ancestry” in females. “Ancestry” was also found to be almost as important as “BMI” in the model generated for both sexes. “Birthweight category” and “adult socioeconomic status” were additionally identified in all variable plots, but were not as comparatively important to decreasing the MSE.

Regression random forest models performed more variably across the three population subsets for TA bias. Models explained approximately 55% of the variance in male TA bias and 57% of the variance in TA bias for both sexes, but the model for TA bias among females only explained 37% of variance (Table 1). Model *R* ^2^ values were highest for males only (0.671), followed by both sexes (0.522), and then females (0.333).

**FIGURE 4:**
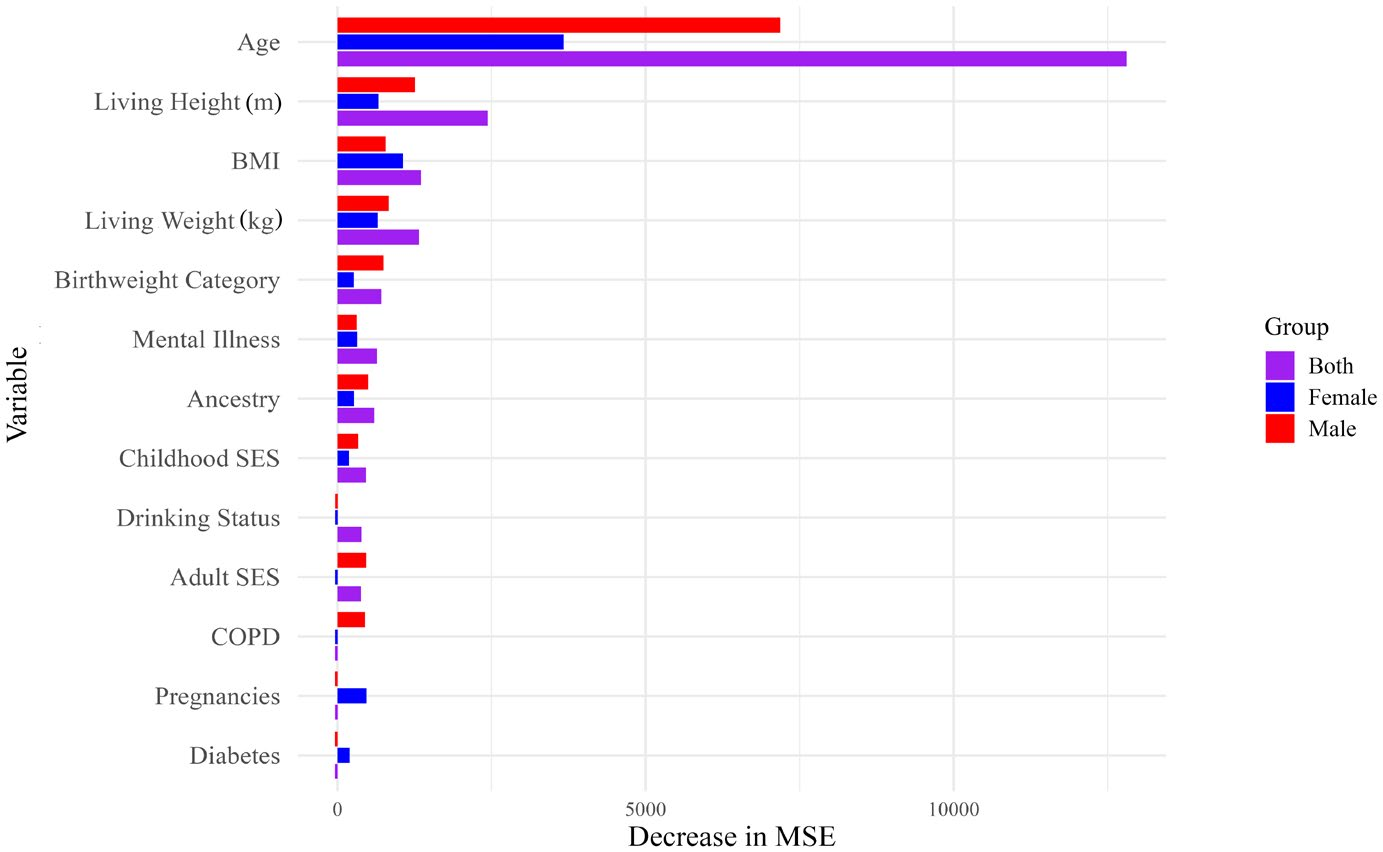
Regression variable importance comparison plot, TA bias. Most important variables to construction of the TA regression forest models for both sexes (purple), males only (red), and females only (blue). Variables are ranked by the influence of that predictor variable on decreasing the overall mean square error (MSE).

Variable rankings for TA bias were similar to those for Suchey–Brooks bias. “Age” was the most important variable across all three plots (Figure 4). For the plot generated for both sexes, this was followed by “living height,” “BMI,” and then “living weight.” For males, “age” was followed by “living height,” “living weight,” “BMI,” and then “birthweight category.” For females, “age” was followed by “BMI,” then “living height,” “living weight,” and “pregnancies.” The remainder of the variables explained very little of the decrease in overall MSE, although “diagnosed mental illness,” “ancestry,” and “childhood socioeconomic status” appeared in all variable importance plots.

Classification random forests were reflective of how well inaccurate age estimates could be predicted, and which variables were most useful in making that prediction. While overall OOB error across all models was relatively low, all models struggled to correctly sort individuals that were not successfully aged by the model (Table 2).

**TABLE 2:** Classification random forest results.

Aging accuracy	Subset	OOB error	Training output description	Test output description
Suchey–Brooks	Males only	26.03	1 of 1 overaged individuals is misclassified as “successfully captured” (class error = 100%); 4 of the 51 successfully aged individuals are misclassified as “underaged” (class error = 7.8%); 13 of the 21 underaged individuals are misclassified as “successfully captured” (class error = 62%).	0 of 0 overaged individuals are misclassified; 3 of 16 successfully aged individuals are misclassified as “underaged”; 2 of 9 underaged individuals are misclassified as “successfully captured”
	Females only	8.51	1 of 1 overaged individuals is misclassified as “successfully captured” (class error = 100%); 0 of 43 successfully aged individuals are misclassified (class error = 0%); 3 of 3 underaged individuals are misclassified as “successfully captured” (class error = 100%)	0 of 0 overaged individuals are misclassified; 2 of 16 successfully aged individuals are misclassified as “underaged” and 2 of 16 individuals are misclassified as “underaged”; 0 of 0 underaged individuals are misclassified
	Both	20.83	3 of 3 overaged individuals are misclassified as “successfully captured” (class error = 100%); 1 of 93 successfully aged individuals are misclassified as “overaged” and 5 of 93 individuals are misclassified as “underaged” (class error = 6.5%); 14 of 24 underaged individuals are misclassified as “successfully captured” (class error = 58%)	0 of 0 overaged individuals are misclassified; 1 of 34 successfully aged individuals is misclassified as “overaged,” and 6 of 34 individuals are misclassified as “underaged”; 1 of 7 underaged individuals is misclassified as “successfully captured”
Transition analysis	Males only	23.29	3 of 3 overaged individuals are misclassified as “successfully captured” (class error = 100%); 1 of 40 successfully aged individuals is misclassified as “overaged,” and 9 of 40 individuals are misclassified as “underaged” (class error = 25%); 6 of 30 underaged individuals are misclassified as “successfully captured” (class error = 20%)	0 of 0 overaged individuals are misclassified; 1 of 11 successfully aged individuals is misclassified as “underaged”; 4 of 14 underaged individuals are misclassified as “successfully captured”
	Females only	23.4	1 of 1 overaged individuals is misclassified as “successfully captured” (class error = 100%); 2 of 38 successfully aged individuals are misclassified as “underaged” (class error = 5.3%); 8 of 8 underaged individuals are misclassified as “successfully captured” (class error = 100%)	0 of 0 overaged individuals are misclassified; 2 of 15 successfully aged individuals are misclassified as “overaged,” 2 of 15 individuals are misclassified as “underaged”; 1 of 1 underaged individuals is misclassified as “successfully captured”
	Both	25	5 of 6 overaged individuals are misclassified as “successfully captured” (class error = 83%); 10 of 77 successfully aged individuals are misclassified as “underaged” (class error = 13%); 15 of 37 underaged individuals are misclassified as “successfully captured” (class error = 41%)	0 of 0 overaged individuals are misclassified; 4 of 30 successfully aged individuals are misclassified as “underaged”; 1 of 11 underaged individuals is misclassified as “successfully captured”

*Note*: Summary and descriptions of classification forest results, where overall model accuracy was evaluated based on the out‐of‐bag (OOB error). Class errors for the training and test data are also provided in the description columns.

OOB error for models testing Suchey–Brooks accuracy ranged from 26.03 (males only) to 8.51 for females only (Table 2). The model constructed for both sexes had an OOB error of 20.83. Variable importance plots demonstrated similar variables to those appearing in regression variable importance plots (Figures S1–S3). “Age” was by far the most important variable across both metrics of variable importance for both sexes (Figure S1) and males only (Figure S2). For males, both metrics of variable importance also indicated “living height” and “diagnosed COPD.” For both sexes, “living height,” “living weight,” “biological sex,” and “reported exercise” were highlighted. The remainder of the variables for both sexes exerted little effect on improving the model.

The variable importance plots generated for female Suchey–Brooks accuracy were the first in which age was not ranked highest (Figure S3). “Living weight” and “BMI” were ranked higher than “age” in both variable importance plots, and “dietary pattern,” “ancestry,” and “childhood socioeconomic status” were ranked higher than “age” in the plot, reflecting the mean decrease in accuracy. “Pregnancies,” “living height,” and “adult socioeconomic status” were additionally highlighted as moderately important in both plots.

In classification models generated for TA accuracy, OOB error ranged from 25 for both sexes to 23.29 for males only (Table 2). Like those constructed for Suchey–Brooks accuracy, “age” was the most important variable in the model constructed for both sexes (Figure S4) and for males (Figure S5), but “age” was not the most important variable in the mean decrease accuracy plot for females only (Figure S6). “Pregnancies,” “living height,” and “diagnosed mental illness” were all ranked higher for females, and appeared in both variable importance plots. “BMI” and “cannabinoid use” were also ranked comparatively highly in both plots.

After “age,” “living height,” and “living weight” were most important in the males‐only TA classification model (Figure S5). Neither had a strong influence on overall model accuracy, and the remainder of the variables were also not shown to affect model success strongly. In the variable importance plots for both sexes, age was followed by living height (Figure S4). The remainder of the lifestyle variables was found to be much less important to overall model accuracy.

## 4 DISCUSSION

### 4.1 Biological sex

The only statistically significant pattern established was between biological sex and TA age estimation bias. Male individuals were typically underaged relative to females of the same age, and female age estimates had an increased heteroskedasticity relative to males (Figure 2). It is therefore likely that biological sex exerts an impact on age estimates produced by ABDOU within the NMDID sample. While Simon and Hubbe's [32] validation study evaluating ADBOU found that there was an absence of sex‐based patterning across age estimates, Kim and Algee‐Hewitt [51] found that point estimates of age generated from pubic symphysis data tended to overage females relative to males, as has been observed here.

Random forest modeling supports the sex‐dependent statistically significant difference in TA aging bias. The regression forest generated for female‐only TA bias explained the least variance of all regression forests: while all other models explained approximately 50% of the variance or greater, only 37% of the variance in TA bias among female individuals was explainable (Table 1).

Despite sex‐based patterning, however, there is no data to suggest that ADBOU creates less accurate female estimates than male estimates; instead, univariate results only suggest that transition analysis produces older age estimates for female individuals relative to males of the same age on average (Figure 2). Random forest results additionally indicate that female age estimates tend to be more variable with increasing age compared to males, and less explainable by age alone. This could be due to an underlying physiological difference, such as an earlier start to bone loss in females relative to males [52], or the effect of pregnancy. While pregnancy was not shown to have a clearly predictable effect on aging bias, it was ranked higher in importance than most lifestyle variables (with the exception of “age” and body size metrics) for Suchey–Brooks and TA bias regression forests (Figures 3 and 4). This indicates that it likely does exert some effect on age‐related symphyseal morphology, although this effect may not be consistent between individuals or across the lifecourse. This also supports existing literature, as biological sex and pregnancy have been implicated in pubic symphysis age estimation inaccuracy previously by Lottering et al. [26], Kim and Algee‐Hewitt [51], Berg [52], Suchey [53], and Suchey and Katz [54].

### 4.2 Body size

Although age estimation bias was not found to be statistically significant between BMI groups using univariate testing, body size metrics were consistently identified as important in the construction of both regression and classification models. In most models, only “age” was found to be more important than either “BMI,” “living height,” “living weight,” or a combination of the three (Figures 3, 4, S1 and S4–S6). In the classification random forest generated for Suchey–Brooks accuracy with respect to females, “living weight” and “BMI” were found to be more influential in predicting inaccurate age estimates than even “age” (Figure S3).

However, it is important to note that regression random forests were unable to explain approximately half of the variation in aging bias (Table 1), and that classification models generally failed to make reliable predictions on both training and testing data (Table 2). Collectively, this suggests that while weight and BMI do exert an impact on age estimation, this effect is not uniform or reliably predictable, and cannot be meaningfully operationalized in forensic age estimates.

It is possible that body size does not affect skeletal age uniformly across age groups, confounding its detection in univariate analyses while explaining its importance in multivariate models in combination with chronological age. While the impact of obesity on bone health is generally understood to be poor [17, 23, 55], it is also understood that low BMI may increase fracture risk, especially in the hips, and precipitate loss of bone mineral density in aging individuals [56, 57]. This has been shown to especially be relevant after the age of 40, and especially in individuals with a history of being underweight [57]. Low BMI has also been linked to bone deterioration and pathologies like osteoporosis and sarcopenia [57, 58].

Random forest results may aid in clarifying earlier discordance between previous studies of body size and age. Merritt [17, 22] found that taller, overweight individuals with a higher BMI are more likely to be overaged, whereas shorter, underweight individuals are consistently underaged when applying the Suchey–Brooks method. In a study of pubic symphyseal CT scans, Merritt [18] again concluded a tendency to overage both males and females of a higher body size. Wescott and Drew [23], however, found no statistically significant differences in the reliability of the Suchey–Brooks method when applied to normal and obese BMI groups. The present study, which suggests that the pubic symphysis is not uniformly impacted by body size, but supports a physiological link between body size and skeletal senescence, may explain the discrepancy between Merritt [17, 18, 22] and Wescott and Drew [23]. Results suggest that body size exerts an influence on skeletal age as previously hypothesized, but this relationship is likely more complex than asserted by Merritt [17, 18, 22].

### 4.3 Other potential influences

Other lifestyle variables did demonstrate a minor influence on aging bias and accuracy. Metrics of socioeconomic status, “ancestry,” and “birthweight category” appeared most important most frequently (Figures 3, 4 and S1–S6). “Ancestry” could be indicative of a genetic or sociocultural influence on aging, whereas metrics of socioeconomic status and birthweight might be suggestive of a link between inequality and generalized aging.

Childhood socioeconomic status and birthweight have notably been used as proxies for early life stress within the Developmental Origins of Health and Disease Hypothesis (DOHaD) framework. DOHaD, conceptualized first by Barker et al. [59], investigates links between early life physiological stress and later disease susceptibility and mortality in adulthood. Research in both clinical and bioarchaeological settings has demonstrated links between primary environments and health outcomes in adulthood [60, 61], most notably for cardiovascular disease, diabetes, certain cancers, and obesity [62]. Other pathologies like osteoporosis, mental illness, and pulmonary diseases have also been associated with higher‐stress early life environments [63]. Links between physiological stress and skeletal aging, however, have been subject to limited investigation. The sole paper identified in the literature studying potential impacts of physiological stress on the accuracy of age‐at‐death estimations was prepared by Simon et al. [39], using the Hamann–Todd Osteological Collection. While no correlations were established, this may be due to the relatively low impact of physiological stress on age. The results of the random forest models presented here indicate that physiological stress during early life, as evidenced by socioeconomic status and birthweight, could be marginally influential on skeletal senescence in the pubic symphysis, but is likely overshadowed by more dominant influences.

Additional research is necessary to clarify relationships between aging and other lifestyle variables. However, the influence of these variables on skeletal aging has been shown here to be complex but minor, and aging methods would likely not be improved by their inclusion.

### 4.4 Study limitations

The first limitation of this study concerns the nature of the lifestyle data itself. Many of the variables contained in NMDID metadata were self‐reported, and many others lack the desired level of descriptive detail. For individuals associated with information regarding reported exercise, there was often minimal detail concerning specific activities, their duration, and their frequency. Additionally, sensitive metadata such as drug use and alcohol consumption patterns may not be reported truthfully, potentially obscuring relationships [64, 65]. It is possible that the inclusion of more detailed information might improve explanations of variability in aging bias, as only approximately half of the variability was able to be accounted for by random forests (Table 1).

It is also unclear if some patterning identified by the random forest algorithms is a consequence of random variation within the dataset. The most influential variables for age estimation accuracy are consistent between both Suchey–Brooks and TA models, and agree with previously published literature. Less important repeatedly identified variables, such as socioeconomic status and birthweight, have a physiological grounding in clinical literature that may explain their effect on the accuracy of aging estimates. However, beyond these variables, the ranking of variable importance lacks uniformity, and many categories within variables may be rare within the sample population. It is also important to note that many lifestyle variables exert influence on each other, and analyzing these variables within a smaller sample may hide some complex interactions in the data that would be more visible in a larger sample population.

The relatively small sample size of this study may also explain high class error rates in classification forests. The majority of age estimates accurately captured chronological age for both Suchey–Brooks and TA, and of individuals that fell outside of expected ranges, the majority were underaged rather than overaged. Overaged individuals were therefore comparatively very underrepresented in the training and testing data.

This research project should therefore only be viewed as a pilot study for additional investigations into the impacts of confounding variables on skeletal aging rates. Additional studies that incorporate more individuals, especially those who are incorrectly aged by skeletal aging estimates, should be conducted in order to verify patterns with respect to such variables.

## 5 CONCLUSIONS

This study enhances a growing body of research that seeks to understand the contribution of demographic and lifestyle variables to inaccuracies in skeletal age estimation. Age estimation bias observed in the outcomes of the ABDOU method corroborates previous research suggesting that there is greater variability in pubic symphysis female age estimates as compared to male [51, 53, 54]. Overall, regression random forest modeling indicated that a wide range of different variables, including both those identified previously in the literature and investigated here for the first time, do likely exert an influence on skeletal aging. However, findings suggest that this influence is comparatively small, nonuniform, and unlikely to greatly influence the accuracy of age estimation from the pubic symphysis.

The findings of this study support a multifaceted etiology for skeletal aging, which is strongly related to chronological age. While body size, pregnancy, and socioeconomic status also may influence the accuracy of age estimation methods, there is a high degree of variability in how these lifestyle variables impact skeletal aging. Similarities between the variables determined to influence age assessment accuracy from the Suchey‐Brooks and ADBOU methods imply that the influence of lifestyle variables is intrinsic to the process of aging of the public symphysis, rather than an artifact of the mathematical approach used to predict age.

As the vast majority of individuals in this sample were correctly aged by both the Suchey–Brooks method and ADBOU, and lifestyle variables did not contribute significantly to age estimation accuracy, we conclude that incorporating considerations of any one or combinations of lifestyle variables would not improve age estimation methods beyond their current capabilities. The ability to investigate and ultimately exclude the potential confounding effects of lifestyle variables on age estimation accuracy, as has been possible here, is highly beneficial for forensic casework, where lifestyle variables of decedents are usually unknown or challenging to accurately reconstruct.

## FUNDING INFORMATION

Marshall Scholarship Thesis Grant, University of Sheffield Department of Archaeology Thesis Grant.

## CONFLICT OF INTEREST STATEMENT

The authors have no conflicts of interest to report.

## Supporting information


Figure S1.

Table S1.

## Data Availability

The data that supports the findings of this study are available in the supplementary material of this article.
