# Supplementary material for: Examining the influence of lifestyle variables on the accuracy of skeletal age estimation via the pubic symphysis
Source: J Forensic Sci. 2025 Nov 28;71(2):656–67. doi: 10.1111/1556-4029.70240 (PMC12967697; doi:10.1111/1556-4029.70240)
Supplement: Supplementary file 1 — Figure S1. [file JFO-71-656-s001.docx]

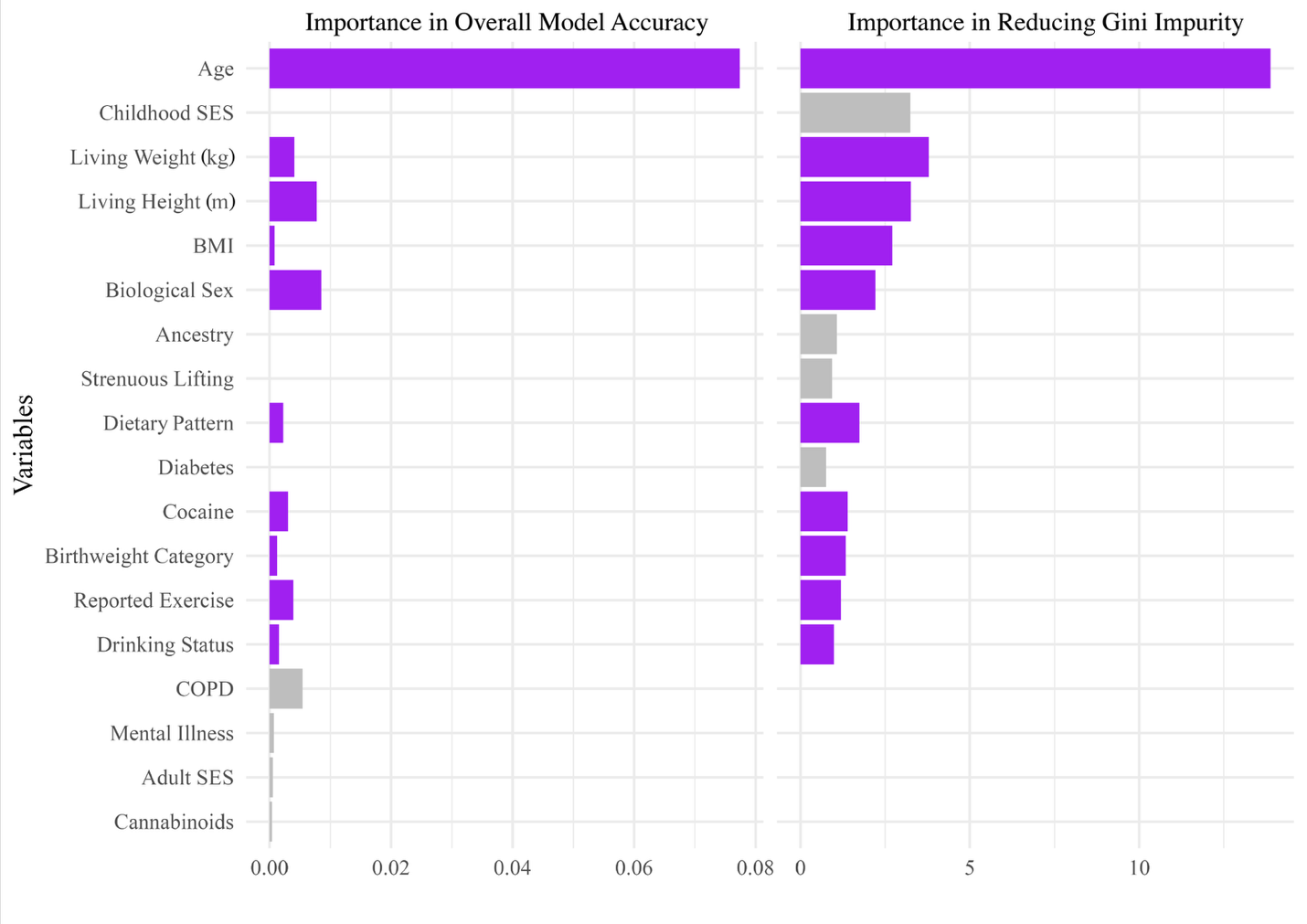


FIGURE S1 **Classification variable importance plots for both sexes, Suchey-Brooks accuracy.**

Most important variables to construction of the Suchey-Brooks classification forest model for both sexes. Variables in each plot are ranked by the importance of each predictor variable on overall accuracy of the model (left) and reducing Gini impurity (right). Variables highlighted by both metrics of variable importance are depicted in purple, whereas variables only appearing by one variable importance plot for that model are depicted in grey.


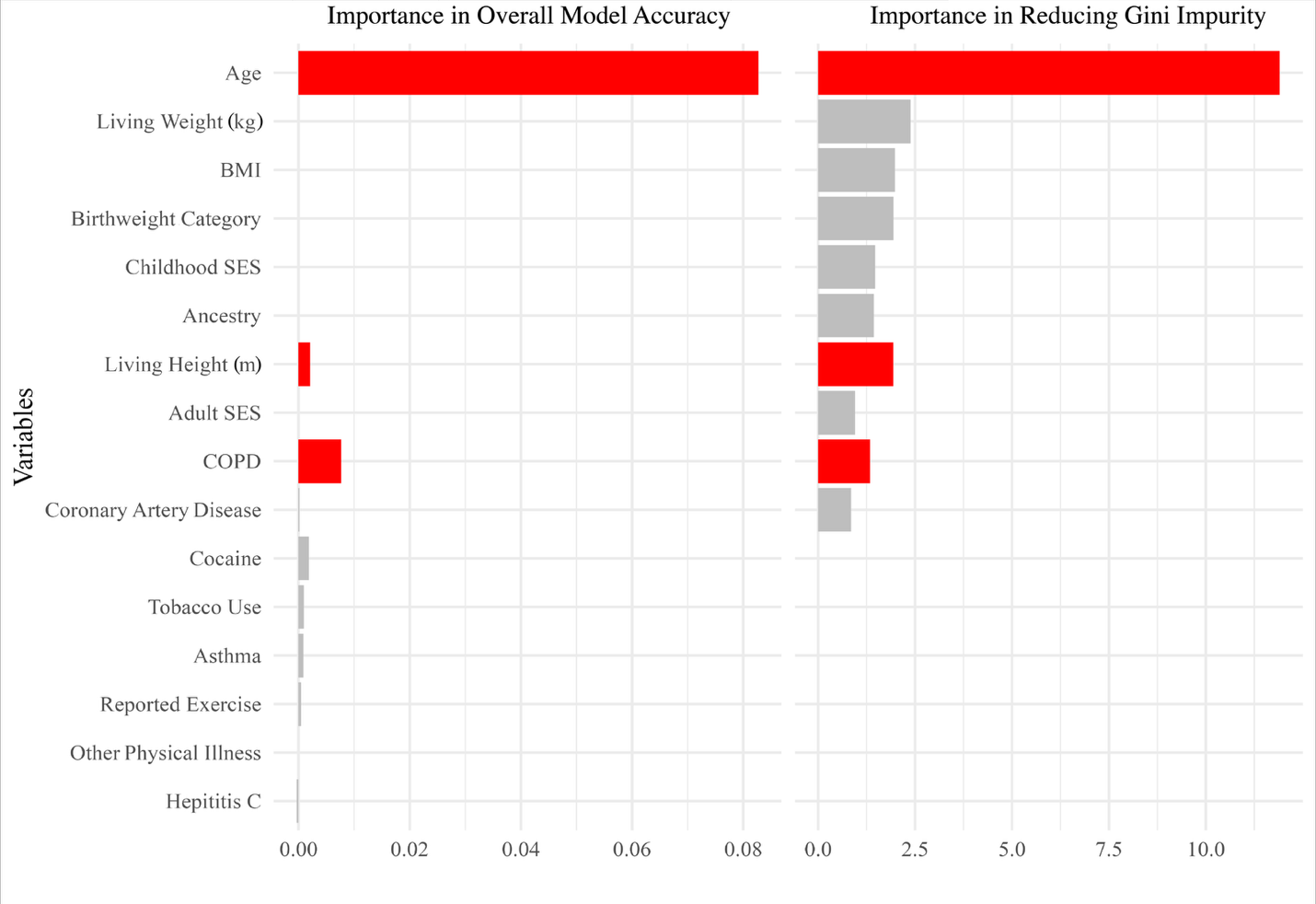
FIGURE S2 **Classification variable importance plots for males only, Suchey-Brooks accuracy.**

Most important variables to construction of the Suchey-Brooks classification forest model for males only. Variables in each plot are ranked by the importance of each predictor variable on overall accuracy of the model (left) and reducing Gini impurity (right). Variables highlighted by both metrics of variable importance are depicted in red, whereas variables only appearing by one variable importance plot for that model are depicted in grey.


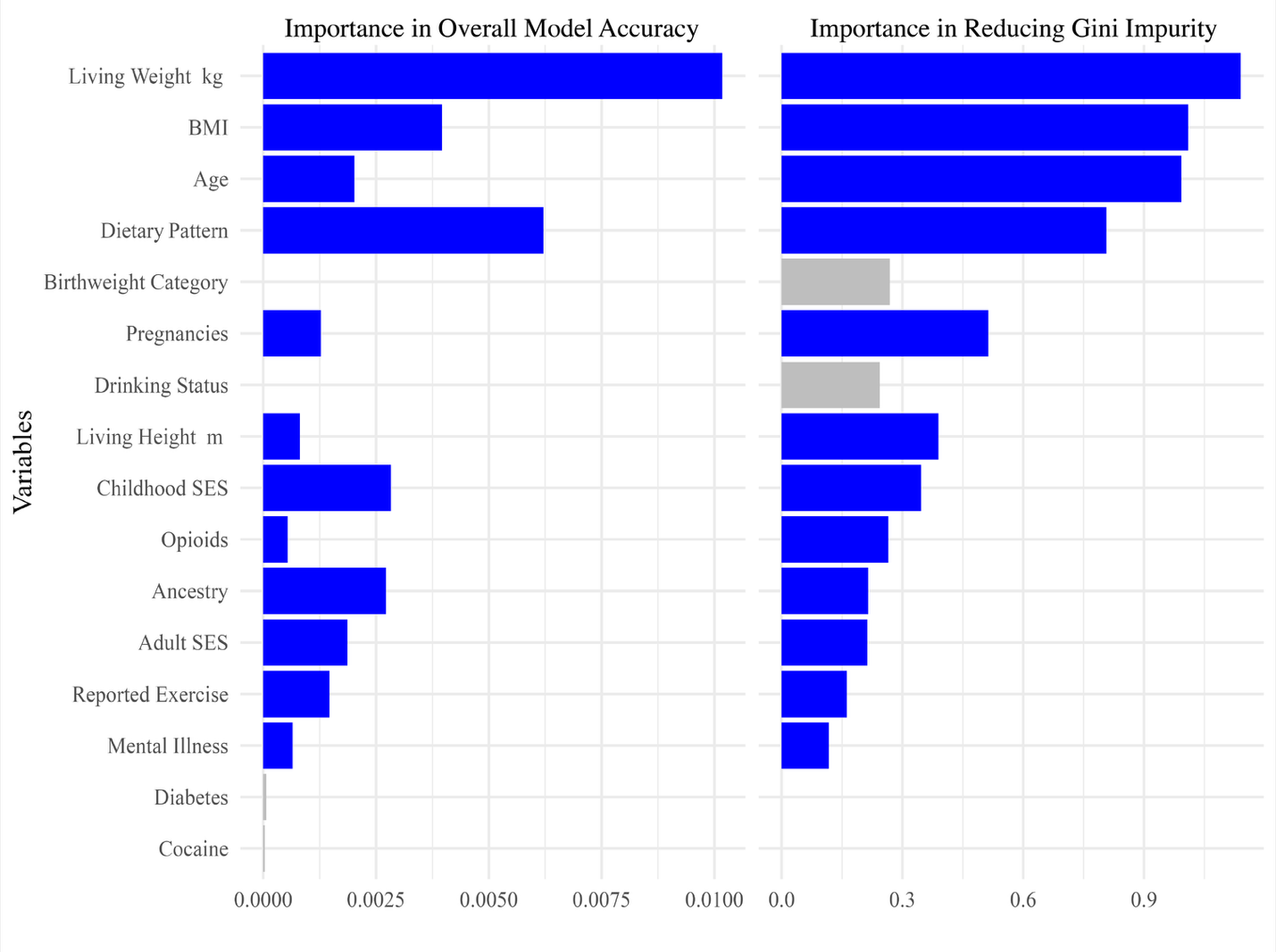


FIGURE S3 **Classification variable importance plots for females only, Suchey-Brooks accuracy.**

Most important variables to construction of the Suchey-Brooks classification forest model for females only. Variables in each plot are ranked by the importance of each predictor variable on overall accuracy of the model (left) and reducing Gini impurity (right). Variables highlighted by both metrics of variable importance are depicted in blue, whereas variables only appearing by one variable importance plot for that model are depicted in grey.


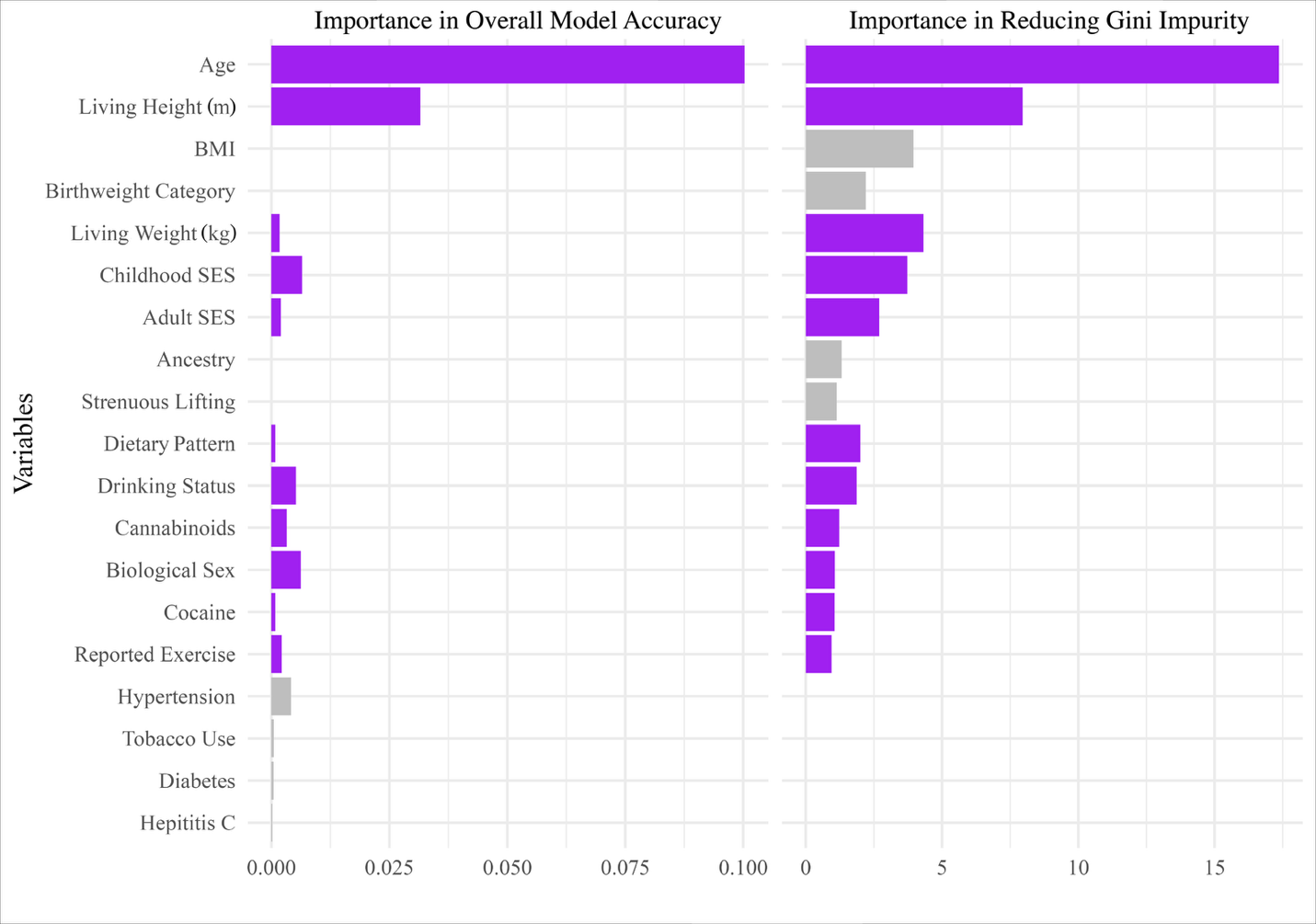


FIGURE S4 **Classification variable importance plots for both sexes, TA accuracy.**

Most important variables to construction of the TA classification forest model for both sexes. Variables in each plot are ranked by the importance of each predictor variable on overall accuracy of the model (left) and reducing Gini impurity (right). Variables highlighted by both metrics of variable importance are depicted in purple, whereas variables only appearing by one variable importance plot for that model are depicted in grey.


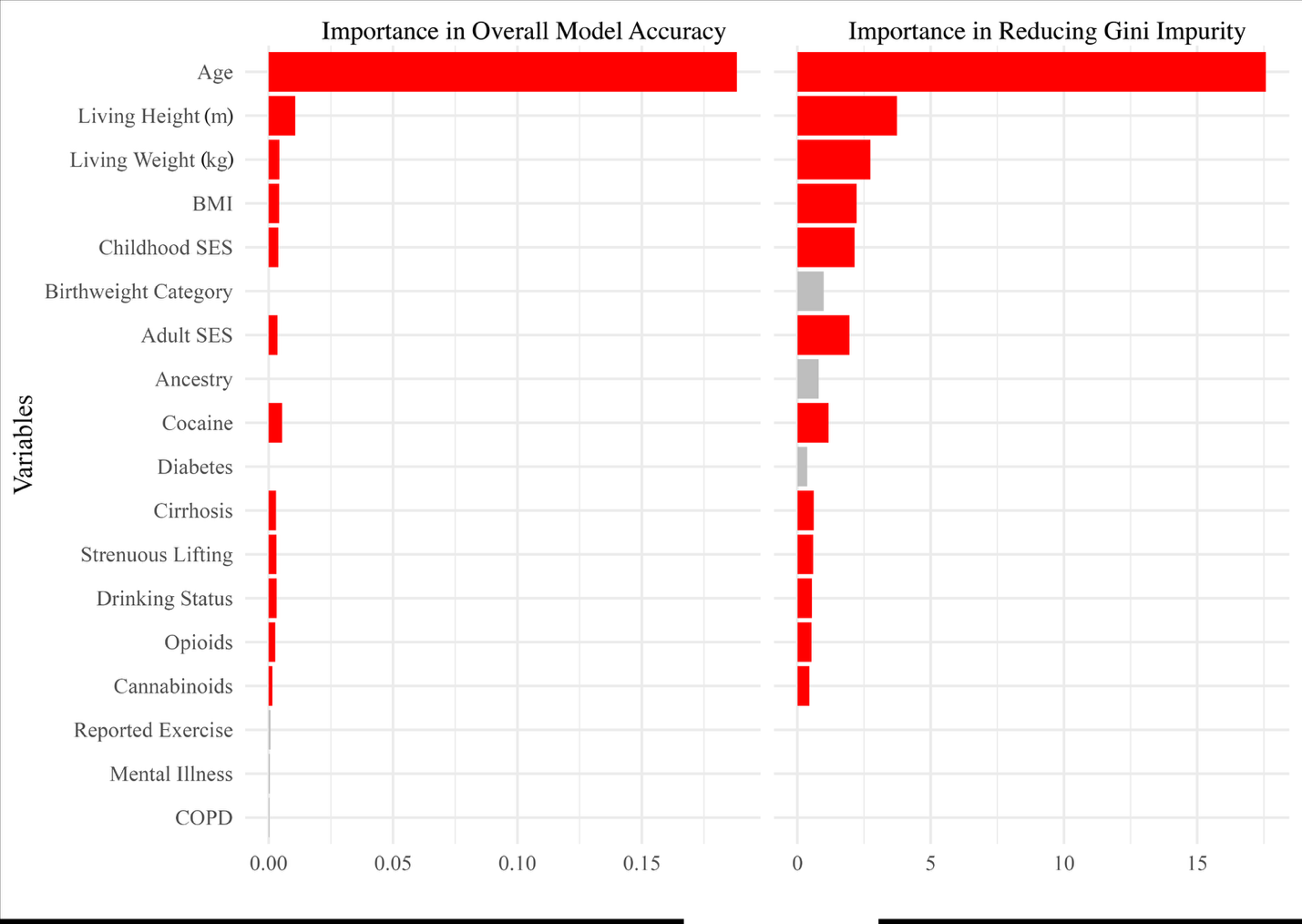


FIGURE S5 **Classification variable importance plots for males only, TA accuracy.**

Most important variables to construction of the TA classification forest model for males only. Variables in each plot are ranked by the importance of each predictor variable on overall accuracy of the model (left) and reducing Gini impurity (right). Variables highlighted by both metrics of variable importance are depicted in red, whereas variables only appearing by one variable importance plot for that model are depicted in grey.


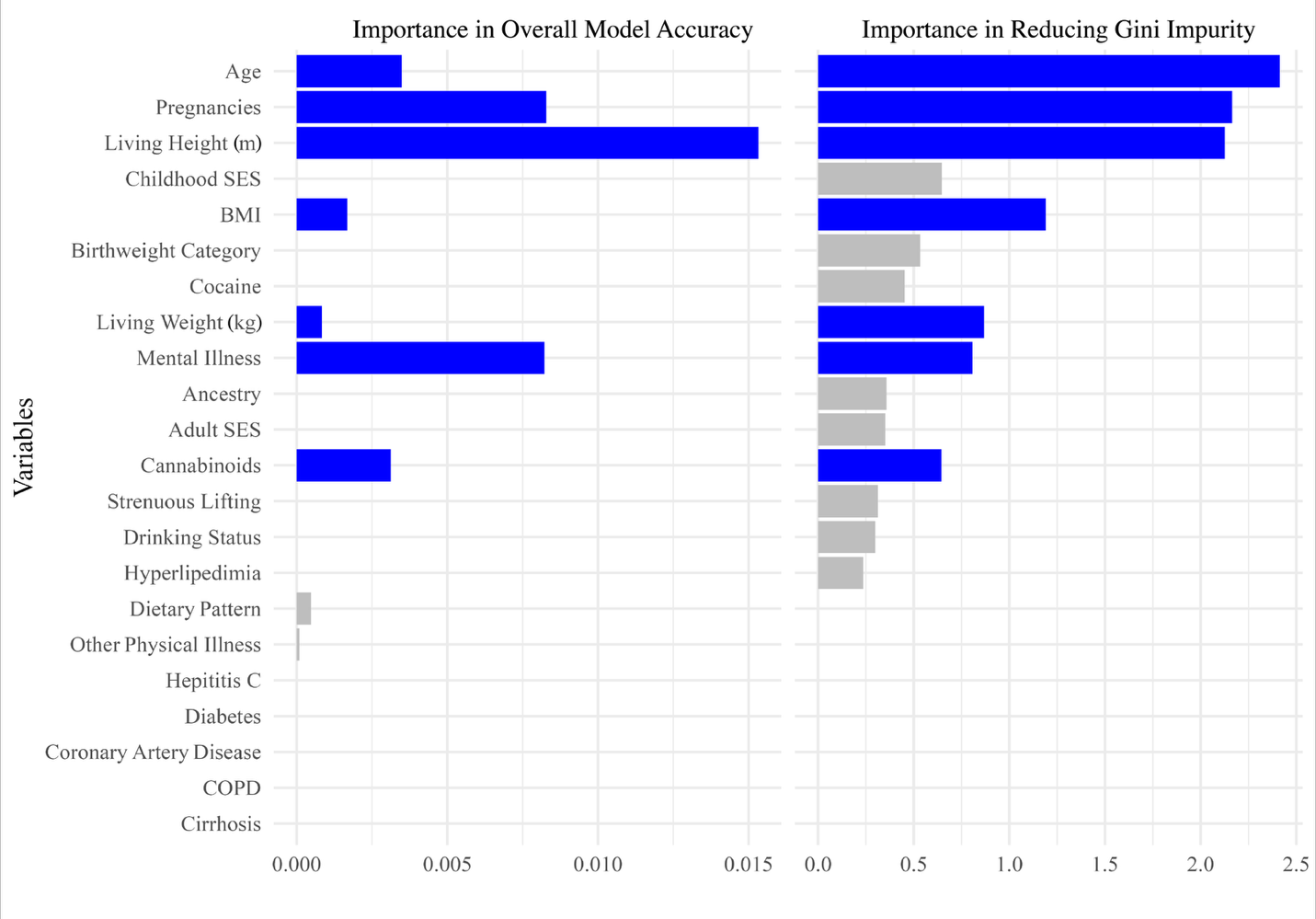


FIGURE S6 **Classification variable importance plots for females only, TA accuracy**.

Most important variables to construction of the TA classification forest model for females only. Variables in each plot are ranked by the importance of each predictor variable on overall accuracy of the model (left) and reducing Gini impurity (right). Variables highlighted by both metrics of variable importance are depicted in blue, whereas variables only appearing by one variable importance plot for that model are depicted in grey.
